# Leukoencephalopathy with brain calcifications and cysts (Labrune syndrome) case report: diagnosis and management of a rare neurological disease

**DOI:** 10.1186/s12883-021-02531-y

**Published:** 2022-01-05

**Authors:** Michelle Paff, Nardin Samuel, Noor Alsafwani, Darcia Paul, Phedias Diamandis, Seth A. Climans, Walter Kucharczyk, Mandy Yi Rong Ding, Andrew F. Gao, Andres M. Lozano

**Affiliations:** 1 Department of Neurological Surgery, University of California Irvine, Irvine, CA USA; 2 Division of Neurosurgery, Department of Surgery, University Health Network, Toronto, ON USA; 3 Laboratory Medicine Program, University Health Network, Toronto, ON USA; 4 Department of Pathology, College of Medicine, Imam Abdulrahman Bin Faisal University (IAU), Dammam, Saudi Arabia; 5 Division of Neurology, Department of Medicine, University Health Network, Toronto, ON USA; 6 Joint Department of Medical Imaging, University Health Network, University of Toronto, Toronto, ON USA

**Keywords:** Labrune syndrome, Leucoencephalopathy, Intracranial cysts, Intracranial calcifications, Case report

## Abstract

**Background:**

Leukoencephalopathy with brain calcifications and cysts (LCC; also known as Labrune syndrome) is a rare genetic microangiopathy caused by biallelic mutations in *SNORD118*. The mechanisms by which loss-of-function mutations in *SNORD118* lead to the phenotype of leukoencephalopathy, calcifications and intracranial cysts is unknown.

**Case presentation:**

We present the histopathology of a 36-year-old woman with ataxia and neuroimaging findings of diffuse white matter abnormalities, cerebral calcifications, and parenchymal cysts, in whom the diagnosis of LCC was confirmed with genetic testing. Biopsy of frontal white matter revealed microangiopathy with small vessel occlusion and sclerosis associated with axonal loss within the white matter.

**Conclusions:**

These findings support that the white matter changes seen in LCC arise as a consequence of ischemia rather than demyelination.

## Background

Leukoencephalopathy with brain calcifications and cysts (LCC), first described by Labrune et al. in 1996 [1], is a rare autosomal recessive genetic disorder caused by biallelic mutations in Small Nucleolar RNA, C/D Box 118 (*SNORD118)*, a non-protein-coding small nucleolar RNA gene on chromosome 17p13.1 [2]. The disease may present with seizures, cerebellar ataxia, or extrapyramidal symptoms and may progress to cognitive decline, brainstem dysfunction, and quadriplegia [1, 3–5]. While the majority of patients present in childhood or young adulthood, multiple instances of late, adult-onset LCC have been reported in the literature [3–10], with the latest reported presentation occurring at 70 years [11]. Neuroimaging shows a characteristic triad of diffuse white matter hyperintense signal on T_2_-weighted imaging, cerebral calcifications, and parenchymal cysts [1]. The most prominent pathological feature is cerebral microangiopathy, with associated gliosis, microhemorrhage, and cerebral calcification [2].

While the genetic origin of LCC has been elucidated, the mechanisms leading to the phenotype of the disease remain obscure. Presumably, sclerosis and occlusion of small vessels causes ischemia, which then leads to downstream pathologic changes. The origin of myelin pallor for which LCC is named is unclear since demyelination has not previously been found in histologic specimens. In this report, we present the pathologic findings from a 36-year-old woman with imaging findings of leukoencephalopathy, cerebral calcifications, and multiple parenchymal cysts. Our case demonstrates that myelin pallor is associated with axonal loss, supporting an ischemic rather than demyelinating mechanism for the white matter changes characteristic of LCC.

## Case presentation

**Fig. 1:**
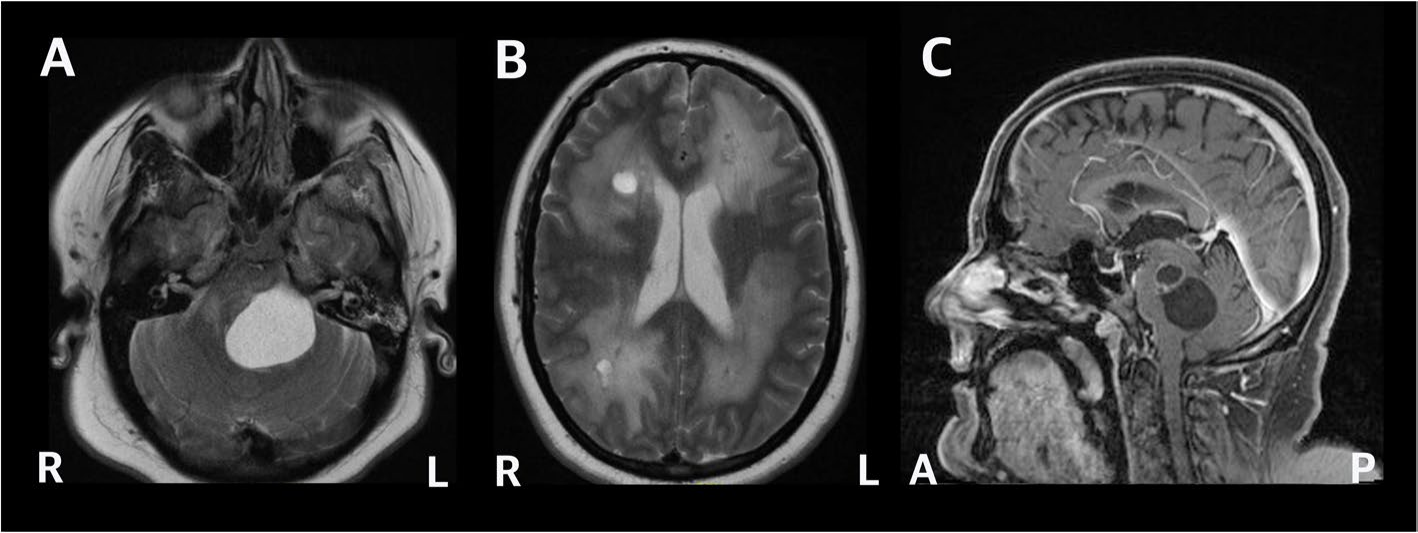
Magnetic resonance imaging (MRI) demonstrating LCC intracranial pathology. T2-weighted axial images demonstrated a cystic lesion in the posterior fossa exerting mass effect upon the brainstem (**A**) and diffuse T2-hyperintense signal change in the hemispheric white matter with periventricular cystic lesions (**B**). T1-weighted sagittal images showed the large posterior fossa cyst expanding from the dorsal pons and compressing the fourth ventricle (**C**)

**Fig. 2:**
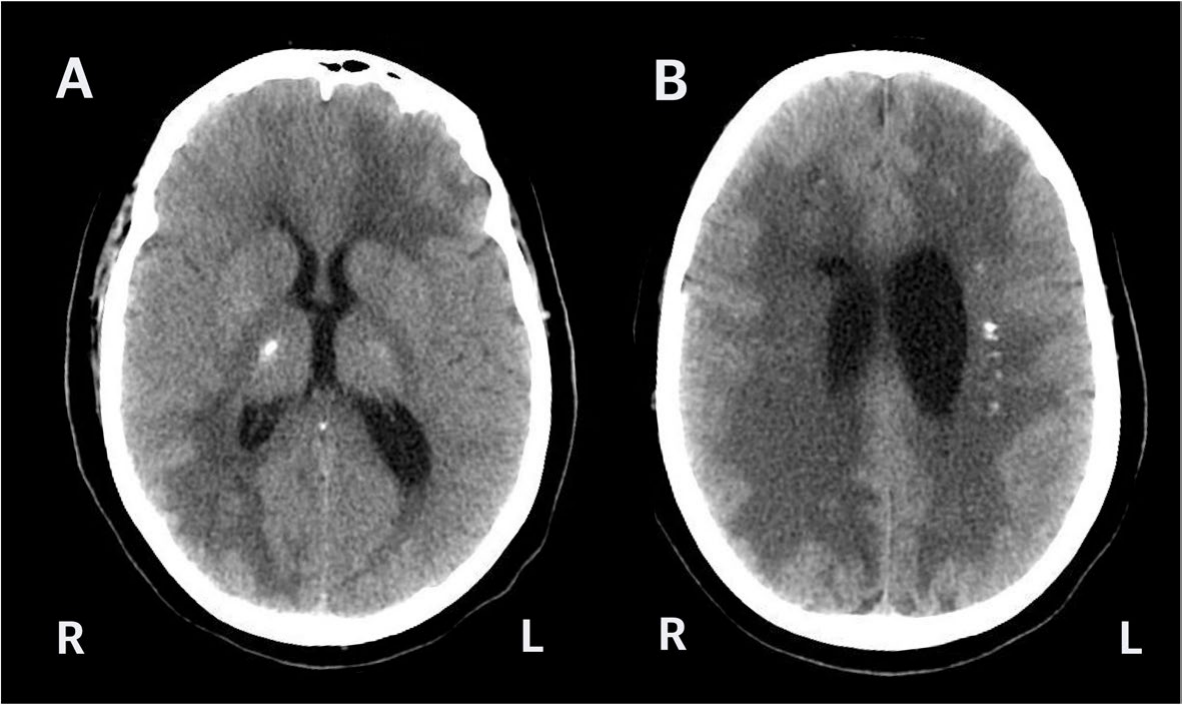
Computed tomography (CT) axial images. Calcifications were seen in the right thalamus (**A**) bilaterally within the cerebral white matter, here shown in the left centrum semiovale (**B**). The cerebral white matter also showed diffuse hypoattenuation (**B**)

A 36-year-old woman with type 2 diabetes mellitus and morbid obesity presented with progressive balance disturbance over several months. She was born premature to healthy non-consanguineous parents, and had an unremarkable family history. On examination, she was alert, oriented, and conversant. She had normal fundoscopy. There were no gross sensory or pyramidal motor abnormalities. Evaluation of coordination with finger-to-nose and rapid alternating movements testing revealed marked left-side dysmetria and ataxia. A gadolinium-enhanced magnetic resonance imaging (MRI) study revealed multiple cystic lesions, most notably in the pons and cerebral white matter, and extensive white matter hyperintensity on T_2_-weighted images (Fig. 1). Computed tomography (CT) revealed small calcifications scattered within the centrum semiovale and right thalamus (Fig. 2). The patient did not have symptoms of hydrocephalus or increased intracranial pressure. Various neoplastic, neuroinflammatory, and infectious etiologies were initially considered in the differential diagnosis, although serology was negative. Nevertheless, she was started on empiric antimicrobial therapy for suspected toxoplasmosis per recommendations from infectious disease consultation. To facilitate a timely diagnosis, a stereotactic biopsy of right frontal white matter was performed.

**Fig. 3:**
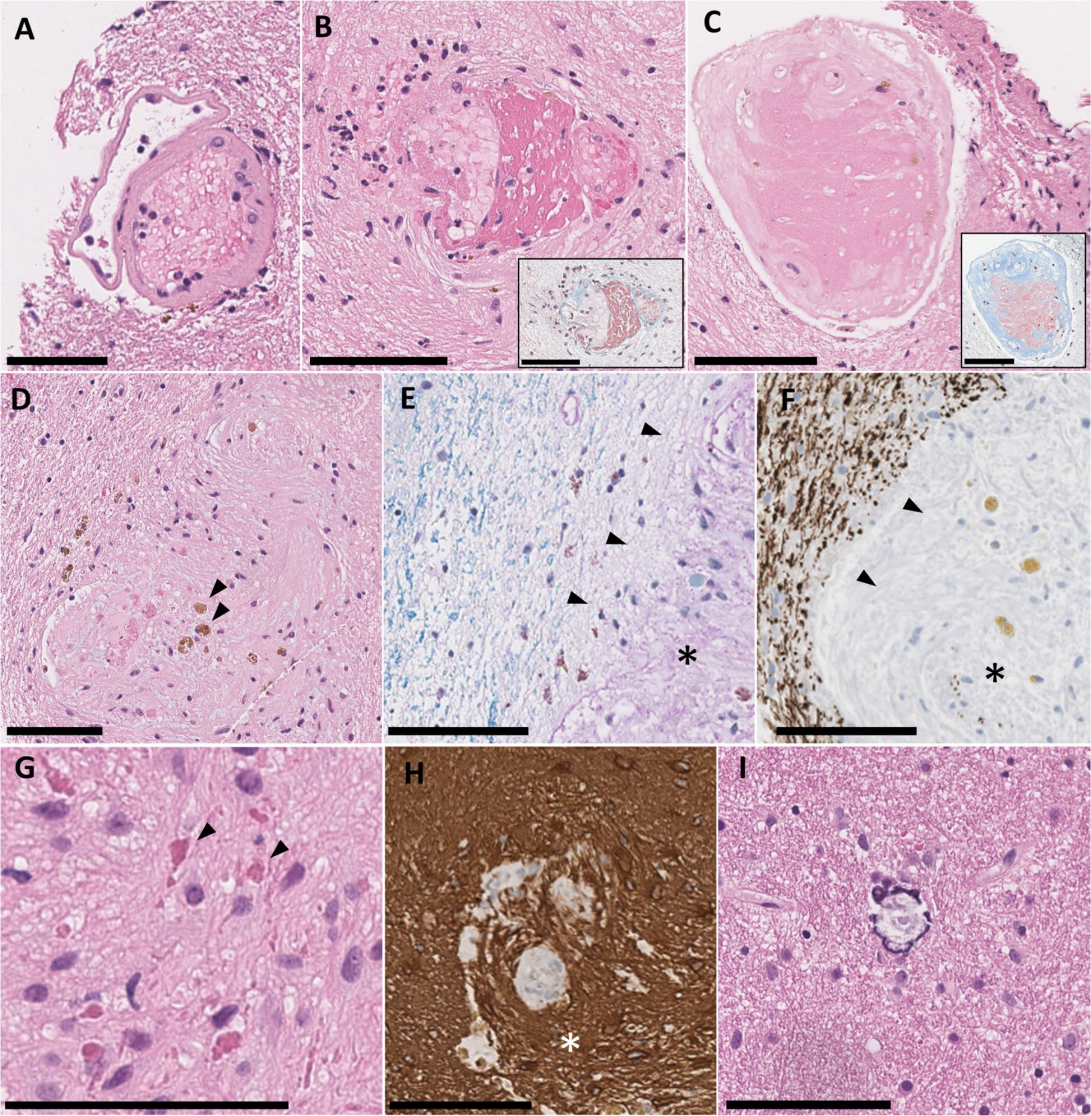
Histopathologic features of LCC. **A**-**D**) **H**&**E**-stained sections showing a spectrum of microangiopathic change, ranging from mild mural hyalinization (**A**) to severe sclerosis with mural deposition of fibrinoid material (**B**,**C**; inset: Martius scarlet blue stain) to complete obliteration (**D**). Perivascular hemosiderin-laden macrophages were a frequent finding (**D**, arrowheads), along with myelin pallor (**E**, LFB/PAS stain, arrowheads; asterisk marks vessel remnant) and axonal loss (**F**, neurofilament immunohistochemistry, arrowheads; asterisk marks vessel remnant). Gliosis was most intense in perivascular areas, with focal formation of Rosenthal fibres (**G**, arrowheads) and highlighted by GFAP immunohistochemistry (**H**, asterick). Small calcospherites were scattered randomly in the white matter (**I**). Scale bars: 100 µm

Histopathologic examination revealed white matter with a spectrum of microangiopathic changes (Fig. 3). The mildest change consisted of dilated vessels with thickened and hyalinized walls. More strikingly, several vessels were severely sclerotic with mural deposition of fibrinoid material with varying degrees of stenosis. Lastly, rare vessels were obliterated, associated with myxoid change in the neuropil. A spectrum of perivascular pathology was also seen. This included frequent hemosiderin-laden macrophages, consistent with chronic microhemorrhage, and intense gliosis with focal formation of Rosenthal fibres. Damaged vessels were often accompanied by perivascular myelin and axonal loss, consistent with ischemic change rather than demyelination (Fig. 3, F). Small calcospherites were scattered throughout the parenchyma randomly. We did not observe any angiomatous proliferations of small vessels. Multidisciplinary review of the pathological findings raised a high index of suspicion for a unifying diagnosis of LCC. Subsequent genetic analysis revealed compound heterozygous n.*9C > T mutation and an n.59 T > C mutation in *SNORD118*, confirming the diagnosis. This is a novel mutation that has not been previously described and impacts the well-conserved box C motif which associates snoRNP proteins. Based on the known spectrum of mutations causing LCC, this variant is predicted to be pathogenic [12]. In particular, an n.59 T > G SNORD118 mutation has been reported in a patient with LCC, providing further evidence for the pathogenticity of the variant in the present case [2].

## Discussion and conclusions

Since the first description of LCC by Labrune and colleagues in 1996 [1], there have been over 100 reported cases worldwide [13]. LCC is cerebral microangiopathy [1, 2] that until recently was hypothesized to belong to a disease spectrum with Coats plus syndrome under the term cerebroretinal microangiopathy with calcifications and cysts (CRMCC) [14, 15]. The latter is characterized by intracranial pathology similar to that seen in LCC, with additional systemic involvement including bilateral exudative retinopathy (Coats disease), skeletal abnormalities, gastrointestinal and hepatic vascular abnormalities, and cutaneous findings such as sparse hair and dystrophic nails [16]. Coats plus syndrome is an autosomal recessive (AR) disease caused by mutations in *CTC1*, which encodes a component of the CTS telomere maintenance complex [17]. When patients without systemic involvement were found to lack *CTC1* mutations, this suggested that LCC was in fact a separate disease [17, 18].

In 2016, Jenkinson and colleagues discovered that LCC is caused by biallelic mutations in the *SNORD118* gene, commonly in a compound heterozygous state [2]. The product of the *SNORD118* gene, box C/D small nucleolar RNA (snoRNA) U8, is a non-protein-coding RNA that plays an essential role in the maturation of specific ribosomal subunits [19]. A recent zebrafish model showed that U8 dysfunction leads to defective central nervous system (CNS) development and rRNA processing defects, and results from the combination of a severe (null) mutation and a milder (hypomorphic) mutation [20]. Although snoRNAs are ubiquitously expressed, loss-of-function mutations in the *SNORD118* gene manifest as a progressive microangiopathy of the CNS through mechanisms that have yet to be fully elucidated.

### Diagnostic features of LCC

The diagnosis of LCC is challenging given the diversity of ages at presentation and its rareness. Neuroimaging features commonly described include diffuse bilateral cerebral white matter T2 hyperintensity, particularly surrounding cysts [21]. Relative sparing of the corpus callosum and subcortical U-fibres has been reported while the posterior fossa white matter is variably affected [22, 23]. Cerebral calcifications are commonly asymmetrically scattered within the cerebral white matter or deep gray nuclei, rarely in the cerebellum, either as small punctate foci or larger confluent areas [11]. Cysts are likewise unevenly distributed throughout the brain parenchyma, most commonly supratentorial but as our case illustrates, may also affect the posterior fossa. Enhancement of the cyst wall and mass effect are common features whereas intracystic hemorrhage has been rarely reported [22]. Diffusion weighted imaging has suggested increased water content within abnormal appearing white matter and MR spectroscopy has suggested energy failure in the cyst wall parenchyma [21, 22]. Together, these findings suggest disruption of the blood brain barrier and white matter edema are features of LCC rather than demyelination, consistent with a microangiopathic pathogenesis [2, 11, 21, 22]. Although the radiologic differential may include various infectious or metabolic disease previously discussed, the triad of diffuse white matter T2 hyperintensity, cerebral calcifications, and parenchymal cysts without retinal or extra-CNS manifestations should suggest the diagnosis of LCC. Exceptionally, systemic involvement has been reported in a genetically confirmed case [23].

Biopsy has been commonly performed in suspected cases of LCC [1–11, 18, 21, 22, 24–40], likely owing to the broad differential diagnosis and the frequent need for neurosurgical intervention to treat mass effect. A pathologically confirmed diagnosis would also obviate the need for empiric antimicrobial or immunomodulatory agents that may be considered while genetic testing is being performed. Previous reports have suggested that biopsy of a cyst wall may carry the highest diagnostic yield [28], but this has simply been the most common site chosen for biopsy and systematic comparison to other sites is lacking. Our case and others illustrate that radiologically abnormal white matter can also yield a diagnostic result [3, 11]. Descriptions of the pathological changes of LCC in genetically confirmed cases are rare, as most reports pre-date the discovery of *SNORD118* mutations [2, 40]. Nevertheless, in clinically and radiologically well-characterized cases, previous authors have described a similar spectrum of microangiopathic changes to our case, with sclerotic and hyalinized vessels occasionally showing fibrinoid deposition, gliosis with Rosenthal fiber formation, chronic microhemorrhage, and parenchymal calcification [1–3, 7, 11, 22, 27, 38, 39]. In contrast, a commonly reported finding we did not observe were “angiomatous” proliferations of thin walled vessels, which have been either the main finding or were present in association with hyaline-sclerosing changes [1, 2, 4–6, 8, 15, 21, 22, 27, 34–40]. This pathologic variability seen in LCC remains to be addressed and may relate to regional variability and location of biopsy, as the cyst wall and more distant white matter may represent different components of the disease. Given the spectrum of microangiopathic change, one could hypothesize that this represents a temporal sequence, with vascular sclerosis, obliteration, ischemia, and cyst formation as the final steps. Conceivably, the angiomatous changes represent a subsequent reaction to hypoxia and would be commonly found in the cyst wall but not more distant white matter. In contrast to the vascular pathology, one consistent finding has been the lack of bona fide demyelination. While several authors have described pallor on myelin staining, there have no reports of active inflammatory demyelination with relative preservation of axons [1, 11, 18, 26, 27, 34, 37]. For the first time, we show that myelin pallor is accompanied by axonal loss, which supports an ischemic rather than demyelinating pathogenesis, consistent with prior radiologic studies. The main pathological substrate for LCC has been consistently shown to be microangiopathy. Hence, leukoencephalopathy may be a misnomer as the white matter changes are likely secondary.

## Data Availability

All data generated or analyzed during this study are included in this published article.

## Abbreviations

LCC: Leukoencephalopathy with brain calcifications and cysts
*SNORD118*: Small nucleolar RNA, C/D box 118
MRI: Magnetic resonance imaging
CT: Computed tomography
CRMCC: Cerebroretinal microangiopathy with calcifications and cysts
AR: Autosomal recessive
snoRNA: Small nucleolar RNA
CNS: Central nervous system
